# Estimation of Total Platelet Count From Peripheral Blood Smear Needs a Correction Factor

**DOI:** 10.7759/cureus.28327

**Published:** 2022-08-23

**Authors:** Nageswar Sahu, Madhusmita Mohanty, Amit K Adhya

**Affiliations:** 1 Pathology, Kalinga Institute of Medical Sciences, Bhubaneswar, IND; 2 Pathology and Laboratory Medicine, All India Institute of Medical Sciences, Bhubaneswar, IND

**Keywords:** multiplication factor, field size, platelet estimation, peripheral smear, total platelet count

## Abstract

Background

Despite many advances in platelet counting by cell counters, the problem of falsely low or falsely high total platelet counts (TPC) is common. Many laboratories estimate platelet count on the peripheral smear to cross-check the platelet counts. However, due to the lack of a standard calculation method, discrepant results are obtained from different laboratories leading to confusion among clinicians. We aimed to formulate a standard estimation method for platelet count on peripheral smear.

Methodology

In the first step (in 100 blood samples), we determined the ratio of the TPC obtained by the automated cell counter and the total number of platelets per oil immersion field (filed size: 0.22 mm) of the corresponding blood smears. The mean of the ratios thus obtained was designated as the “multiplication factor” to be used for visual platelet count estimation on the peripheral blood smear. In the subsequent step, validation of the same was done on another 100 samples. TPC on the peripheral smears of these samples was estimated using the above “multiplication factor” and compared with the corresponding TPC obtained on the automated cell counter.

Results

The “multiplication factor” obtained was 9.4 x 10^3^ in the first set of 100 blood samples. It was used to estimate the platelet value of the second set of 100 blood samples.

Conclusions

We found an excellent agreement between the platelet counts obtained by automated cell counters and the estimation method. We suggest the multiplication factor 9.4 x 10^3^ may be used with correction for microscopic field size to estimate platelet count on peripheral smears. This method is, however, not so reliable for very low platelet counts.

## Introduction

Total platelet count (TPC) is an essential part of a routine hemogram. Most clinical laboratories perform it by automated cell counters. Despite many technological advances, the problem of spurious low or high platelet counts by automated cell counters is common in routine laboratory practice [1,2]. Hence, most laboratories cross-check any abnormal or suspicious platelet values obtained from the cell counters by an alternative method, which includes the estimation of platelet count on the Leishman-stained peripheral blood smear or by manual method of counting platelets in a Neubauer chamber [3].

However, manual platelet count using the Neubauer chamber is cumbersome and time-consuming. It is impractical to use this method in laboratories obtaining large sample sizes. Hence, most laboratories estimate the platelet count on the Leishman-stained peripheral blood smear in cases where the platelet count provided by the automated cell counters needs to be cross-checked. The usual method followed is calculating the average number of platelets per oil immersion field (OIF) and multiplying it with a factor to find the TPC [3]. The multiplication factor reported in the literature varies from 10 x 10^3^ to 20 x 10^3^ [4-8]. This method of platelet count estimation from peripheral blood smear has led to inconsistent results in different laboratories on the same blood sample. This variation in the multiplication factor reported by the different researchers may be because of the use of varied microscopes with different field sizes [3]. We aim to resolve this issue by finding the “multiplication factor” for a commonly used laboratory microscope and propose a formula for correction, which considers the microscope's field size for estimating the platelet count on the peripheral blood smear.

## Materials and methods

We studied 200 consecutive hemogram samples (ethylenediaminetetraacetic acid venous blood) received in the hematology laboratory, which included samples from both inpatients and outpatients of all ages and gender. All samples were analyzed within two hours of collection. Hemolytic samples, clotted samples, or inadequate samples were excluded. Only cases with a fitting curve of platelet histogram were included in the study. Cases with platelet flagging on automated analyzer like platelet clumps, abnormal platelet distribution, or giant platelet and/or cases showing platelet clumps on peripheral smears were excluded.

The 200 samples were divided into two sets of 100 samples each. The first 100 samples (relabeled as 1 to 100) were used to obtain the multiplication factor for estimating the platelet count on peripheral smears. The second set of 100 samples (relabeled as 101 to 200) was used to validate the findings. The automated cell counter used was Beckman Coulter LH750 (Beckman Coulter Inc., Brea, CA). In each case, a peripheral blood smear was prepared and stained with Leishman stain. Three pathologists did an independent estimation. The pathologists were blinded as regards the platelet count obtained on the automated cell counter (PA). All three pathologists used the Olympus FX53 microscope (field number 22) (Olympus Corporation, Tokyo, Japan) for this. On each smear, the numbers of platelets were counted in 10 consecutive OIFs, in the area where the RBCs were just touching each other without any overlapping. The average number of platelets per OIF was obtained by dividing the total number of platelets by 10. The mean of the three observations (obtained by three pathologists) was calculated for each sample (PS). In the first set of samples, the ratio of the platelet count obtained by the automated cell counter (PA1) and the mean value of the number of platelets obtained by visual smear counting (PS1) was calculated for each sample (R = PA1/PS1). Finally, the mean value of "R" was calculated to obtain the multiplication factor (F), to be used for estimating the TPC by the visual smear method. In the second set of samples, in each case, the mean value of the number of platelets obtained by the visual smear counting (PS2) was multiplied by the multiplication factor "F" (calculated from the first 100 samples) to obtain the estimated value of TPC (PE). These values were compared with the platelet counts of these samples obtained on the automated cell counter (PA2).

Analysis of method comparison was done by Pearson's correlation coefficient method and Bland-Altman's 95% limits of agreement method [9]. For this analysis, the automated cell counter method (PA2) was taken as the reference method and visual smear counting (PE) as the testing method.

## Results

A total of 200 samples were included in the study. The age of the patients ranged from one-day-old baby to 83 years. The male-to-female ratio was 3:2.6. Distribution of the TPC in the two sets of samples is given in Table 1.

**Table 1:** Distribution of the total platelet count in the two sets of samples TPC: total platelet count.

	1st set of samples (n = 100): TPC by automated cell counter (PA1)	2nd set of samples (n = 100): TPC by estimation method (PE)	2nd set of samples (n = 100): TPC by automated cell counter (PA2)
Lowest value of TPC (x10^3^/µL)	7	9.3	6.8
Highest value of TPC (x10^3^/µL)	690	656.3	689
No. of samples with TPC > 400 x 10^3^/µL	6	6	7
No. of samples with TPC = 150-400 x 10^3^/µL	26	23	29
No. of samples with TPC < 150 x 10^3^/µL	68	71	64

Calculation of the multiplication factor F

The ratio of PA1 to PS1 range from 5.63 x 10^3^ to 14.71 x 10^3^ with a mean value of 9.42 x 10^3^, standard deviation of 1.43 x 10^3^, standard error of 0.11 x 10^3^, and 95% confidence interval of 9.18 x 10^3 ^to 9.68 x 10^3^. Hence, the desired multiplication factor (F) for estimation of platelet count on smear was 9.42 x 10^3^.

Estimation of platelet count on smears using the multiplication factor

In the second set of 100 samples, the multiplication factor 9.42 x 10^3^ was multiplied by the average number of platelets in the smear per OIF (PS2) to estimate the TPC (PE).

Statistical analysis

**Figure 1:**
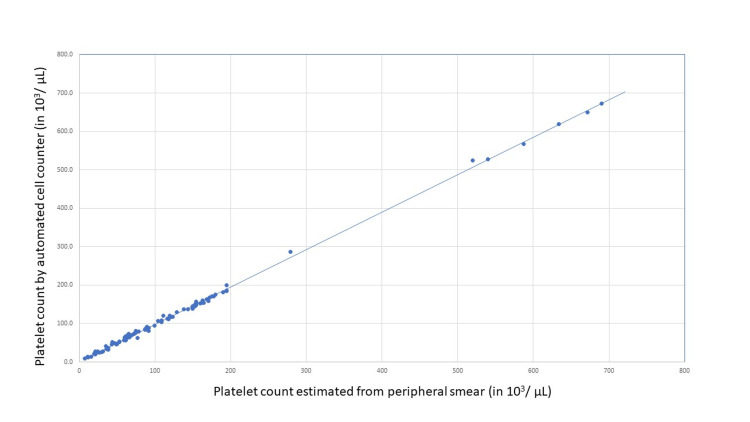
Scatter diagram showing the results of Pearson’s correlation coefficient test applied to compare the platelet counts obtained by the manual calculation method and by the automated cell counter

**Figure 2:**
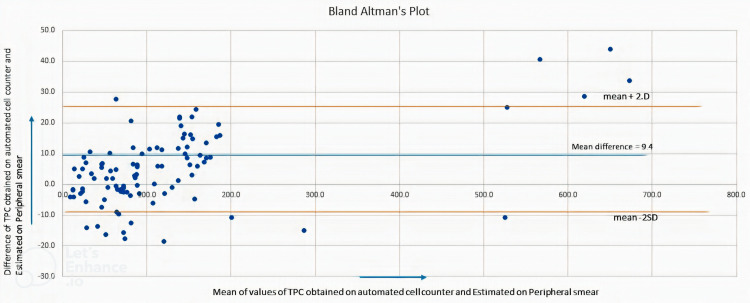
Diagram depicting the results of the Bland-Altman plot Most values are concentrated around the mean and within the 2SD. TPC: total platelet count.

In the second set of 100 samples, the correlation of the TPC values obtained by the estimation method (PE) and by the automated cell counter method (PA2) was done by Pearson coefficient of correlation. The R-value, R^2^ values, and p-values were calculated for various ranges of platelet count (Tables 2, 3). The correlation is also depicted by the scatter diagram (Figure 1). Agreement between the two methods, e.g. automated cell counter and estimation from the peripheral smear, was also assessed by Bland-Altman's method (Figure 2). These results showed an excellent agreement between the two methods when the platelet count is >20 x 10^3^/μL. For samples with TPC < 20 x 10^3^/μL, the estimation method did not show a significant correlation with the automated cell counter.

**Table 2:** Correlation between the two methods in the second set of samples (n = 100)

Total platelet count by automated cell counter	n (100)	Pearson’s correlation coefficient “R-value” (automated cell counter method vs. estimation method)	R^2^ coefficient of determination	P-value
>400 x 10^3^/µL	7	0.9699	0.9407	0.001345
150-400 x 10^3^/µL	29	0.9677	0.9364	<0.00001
<150 x 10^3^/µL	64	0.9692	0.9393	<0.00001
Total	100	0.9971	0.9942	<0.00001

**Table 3:** Correlation between the two methods in the second set of samples with TPC < 150 x 103/µL TPC: total platelet count.

Total platelet count by automated cell counter	n (64)	Pearson’s correlation coefficient “R-value” (automated cell counter method vs. estimation method)	R^2^ coefficient of determination	P-value
50-149.9 x 10^3^/µL	40	0.927	0.8599	<0.00001
20-49.9 x 10^3^/µL	17	0.7135	0.5091	0.001299
<20 x 10^3^/µL	7	0.6084	0.3702	0.147

## Discussion

Accurate platelet count is essential for the proper management of patients. Nowadays, the platelet count is done mainly by an automated cell counter. Most of these cell counters identify platelets based on the particle size. The platelet count obtained from these automated analyzers is usually precise. However, the count can be falsely high in the presence of RBC fragments, bacteria, fungi, lipids, and cryoglobulins. Similarly, the platelet count can be falsely low in the presence of large platelets [1,2]. Hence, in case of any flagging for platelet count by the automated cell counter, the value is cross-checked by alternate manual methods. The International Council for Standardization in Haematology (ICSH) and the International Society for Laboratory Hematology (ISLH) have recommended a method based on platelet/RBC ratio and fluorescent-labeled platelets as the reference method for platelet count estimation [1-3]. However, its high cost prevents its routine use in most laboratories. The most common method applied for cross-checking the automated cell counter report on platelet count is the microscopic inspection of the stained peripheral blood smears and estimating the platelet count from it. Alternatively, the traditional method of manual platelet counts using the Neubauer chamber is employed by some laboratories. But it is a time-consuming, labor-intensive process and is frequently troubled by the presence of artifacts.

Estimating platelet count by microscopic inspection of peripheral smear is a well-established method [1,4]. Despite the recent advances in hematology automation, peripheral blood smear examination remains essential for validating the results of other methods for platelet counting. To date, even the best quality hematology analyzer also cannot replace the peripheral blood smear evaluation [1]. This method counts platelet in consecutive OIFs. The validity of this method was first established by Nosanchuk et al. [4]. It is simple, reliable, cost-effective, and can be used in an emergency as well as in low-resource settings where automated analyzers are unavailable [1,3]. This method has the advantage of identifying platelet clumps/giant platelets/platelet satellitism or the presence of fragmented RBCs [3,10-12]. In addition to the TPC, it helps in assessing additional information like platelet size, granulation, and the phenomenon of platelet aggregation and satellitism [8]. However, it has the disadvantages like marked inter-observer variability in counting the average number of platelets per OIF as noted by Nosanchuk et al. and Gao et al. [1,13]. This variability is usually due to two different reasons. One is the differences in the area of blood smear selected for evaluation. Such variations can be minimized by examining an area where the platelets are regularly distributed and where the red blood cells are in a monolayer at the junction of the body and tail [3]. The second factor responsible for the variability is the variety of available microscopes with varying field numbers leading to a difference in field diameter and field area. This effect can be minimized by adjusting the multiplication factor according to the field diameter, which depends on the field number mentioned in the microscope. Most probably, for this reason, different authors mention different multiplication factors like 15 x 10^3^ or 20 x 10^3^ [5-8,13]. We suggest that the multiplication factor should be modified based on the field number of the microscope. Sudalaimuthu et al. also suggested modified multiplication factors according to the field number of the microscope [3].

An accurate count is crucial for low platelet values as it dictates the clinical decision for platelet transfusion [14]. Hence, clinicians tend to send patient samples to multiple laboratories to confirm the platelet count. In the absence of a uniform standard multiplication factor, different laboratories use different multiplication factors, which leads to confusing variable results obtained from different laboratories and making the clinicians indecisive as regards the requirement of platelet transfusion.

One way to solve this issue is that each laboratory determines its own "multiplication factor." The multiplication factor was found to be 9.4 x 10^3^, with a standard error of ±0.11 x 10^3^. A standard error of 110 platelets/μL is a reasonably acceptable margin for platelet count. Further, we validated this value on another 100 samples, which included low, normal, and high platelet counts.

We found a high degree of correlation and agreement between the TPCs obtained by the automated cell counter and the estimated platelet count on the peripheral smear by multiplying with the factor 9.4 x 10^3^. Laboratories are more likely to cross-check a low platelet count obtained by the automated cell counter rather than a high count. Our results show that the estimated platelet count correlates very well with the automated cell counter values at low platelet counts (20 x 10^3^/μL to 150 x 10^3^/μL) and thus can be used as an adjunct to automated cell counters for confirmation of low platelet counts. However, for TPC values < 20 x 10^3^/μL, the estimation method is not so reliable and must be used with caution. In instances where there is extensive clumping of platelets or platelet satellitism, accurate counting of platelets on peripheral smear may not be possible. In those cases, a direct smear from a finger prick blood sample may be used to avoid the effect of the anticoagulant.

Our results also show a very strong positive correlation between the manual estimation method and the automated cell counter method of platelet counting at normal and high TPC values. Although most laboratories do not routinely employ any alternative method to cross-check normal or high platelet values, the role of platelet estimation on peripheral smear in such situations would be for internal quality assurance. For laboratories participating in quality assurance schemes, platelet estimation on peripheral smear on random samples or samples with low, normal, and high platelet counts could serve as a tool for internal quality assessment.

Counting platelets on the Leishman-stained peripheral blood smear must be done at the junction of body and tail where the RBCs are just touching each other without any overlapping. Proper staining of the smear is essential. Platelets may not be properly visualized in an under-stained smear. Conversely, stain deposits may be mistaken for platelets in an over-stained smear. Care must be taken not to mistake malaria parasites as platelets.

An important reason behind the disparity of the multiplication factor described in various studies may be the difference in the field size of the microscope used. We propose to use the multiplication factor obtained in our study with a correction for the microscope field size. The formula "9.4 x 0.22 x 10^3^/FS" may be used to correct the field size, where FS denotes the field size (in millimeter) of the OIF of the microscope that the laboratory intends to use. Field size is calculated by dividing the field number (written on the eyepiece) by objective magnification. Sudalaimuthu et al. [3] also suggested a multiplication factor very close to our value for use in a microscope with field number 22. We further suggest that more studies should be done using different automated cell counters and microscope models to validate our findings.

## Conclusions

In this study, we highlight the problem of discrepant platelet count values obtained from different laboratories by employing various multiplication factors and suggest a method to reduce such discrepancies. The multiplication factor 9.4 x 10^3^ may be used with correction for microscopic field size to estimate platelet count on peripheral smears. We found a high degree of correlation and agreement between the TPCs obtained by the automated cell counter and estimated platelet count on peripheral smear in cases with platelet counts > 20 x 10^3^/μL. This method is, however, not recommended for very low platelet counts, i.e., < 20 x 10^3^/μL.

## References

[1] Anchinmane VT, Sankhe SV (2019). Utility of peripheral blood smear in platelet count estimation. Int J Res Med Sci.

[2] Baccini V, Geneviève F, Jacqmin H (2020). Platelet counting: ugly traps and good advice. Proposals from the French-speaking Cellular Hematology Group (GFHC). J Clin Med.

[3] Sudalaimuthu M, Rajendran K, Ganapathy S (2017). Modifications to the manual assessment of platelet counts from peripheral blood smears- an essential correction never mentioned before in literature. Indian J Pathol Res Pract.

[4] Nosanchuk JS, Chang J, Bennett JM (1978). The analytic basis for the use of platelet estimates from peripheral blood smears. Laboratory and clinical applications. Am J Clin Pathol.

[5] Webb DI, Parker L, Webb K (2004). Platelet count assessment from peripheral blood smear (PBS). Alaska Med.

[6] Malok M, Tichener EH, Bridgers C, Lee BY, Bamberg R (2007). Comparison of two platelet count estimation methodologies for peripheral blood smears. Clin Lab Sci.

[7] Bajpai R, Rajak C, Poonia M (2015). Platelet estimation by peripheral smear: reliable, rapid, cost-effective method to assess degree of thrombocytopenia. Int J Med Sci Res Pract.

[8] Al-Hosni ZS, Al-Khabori M, Al-Mamari S, Al-Qasabi J, Davis H, Al-Lawati H, Al-Riyami AZ (2016). Reproducibility of manual platelet estimation following automated low platelet counts. Oman Med J.

[9] Altman DG, Bland JM (1983). Measurement in medicine: the analysis of method comparison studies. Statistician.

[10] Marionneaux S, Francisco N, Chan V (2013). Comparison of automated platelet counts and potential effect on transfusion decisions in cancer patients. Am J Clin Pathol.

[11] Kunz D (2001). Possibilities and limitations of automated platelet counting procedures in the thrombocytopenic range. Semin Thromb Hemost.

[12] Lin J, Luo Y, Yao S, Yan M, Li J, Ouyang W, Kuang M (2015). Discovery and correction of spurious low platelet counts due to EDTA-dependent pseudothrombocytopenia. J Clin Lab Anal.

[13] Gao Y, Mansoor A, Wood B, Nelson H, Higa D, Naugler C (2013). Platelet count estimation using the CellaVision DM96 system. J Pathol Inform.

[14] Segal HC, Briggs C, Kunka S, Casbard A, Harrison P, Machin SJ, Murphy MF (2005). Accuracy of platelet counting haematology analysers in severe thrombocytopenia and potential impact on platelet transfusion. Br J Haematol.

